# Supplementary material for: The antimicrobial peptide thanatin disrupts the bacterial outer membrane and inactivates the NDM-1 metallo-β-lactamase
Source: Nat Commun. 2019 Aug 6;10:3517. doi: 10.1038/s41467-019-11503-3 (PMC6684654; doi:10.1038/s41467-019-11503-3)
Supplement: Supplementary file 1 — Supplementary information [file 41467_2019_11503_MOESM1_ESM.pdf]

Supplementary information

The antimicrobial peptide thanatin disrupts the bacterial outer membrane and inactivates the NDM-1 metallo-β-lactamase

Ma et al.

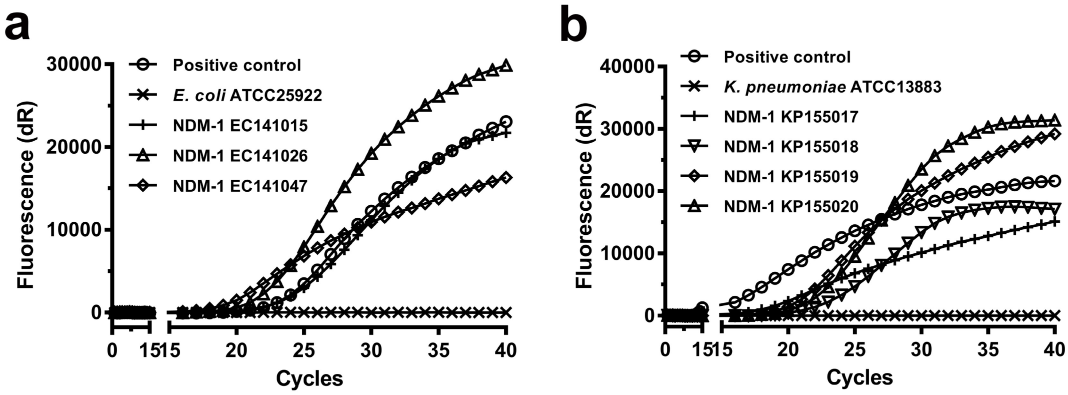

Supplementary Figure 1. Examination of *bla*$_{\text{NDM-1}}$ gene expression. The *bla*$_{\text{NDM-1}}$ gene was detected in clinical isolates of *E. coli* (**a**) and *K. pneumonia* (**b**), with *E. coli* ATCC25922 and *K. pneumonia* ATCC13883 as negative controls. Source data are provided in Source Data file.

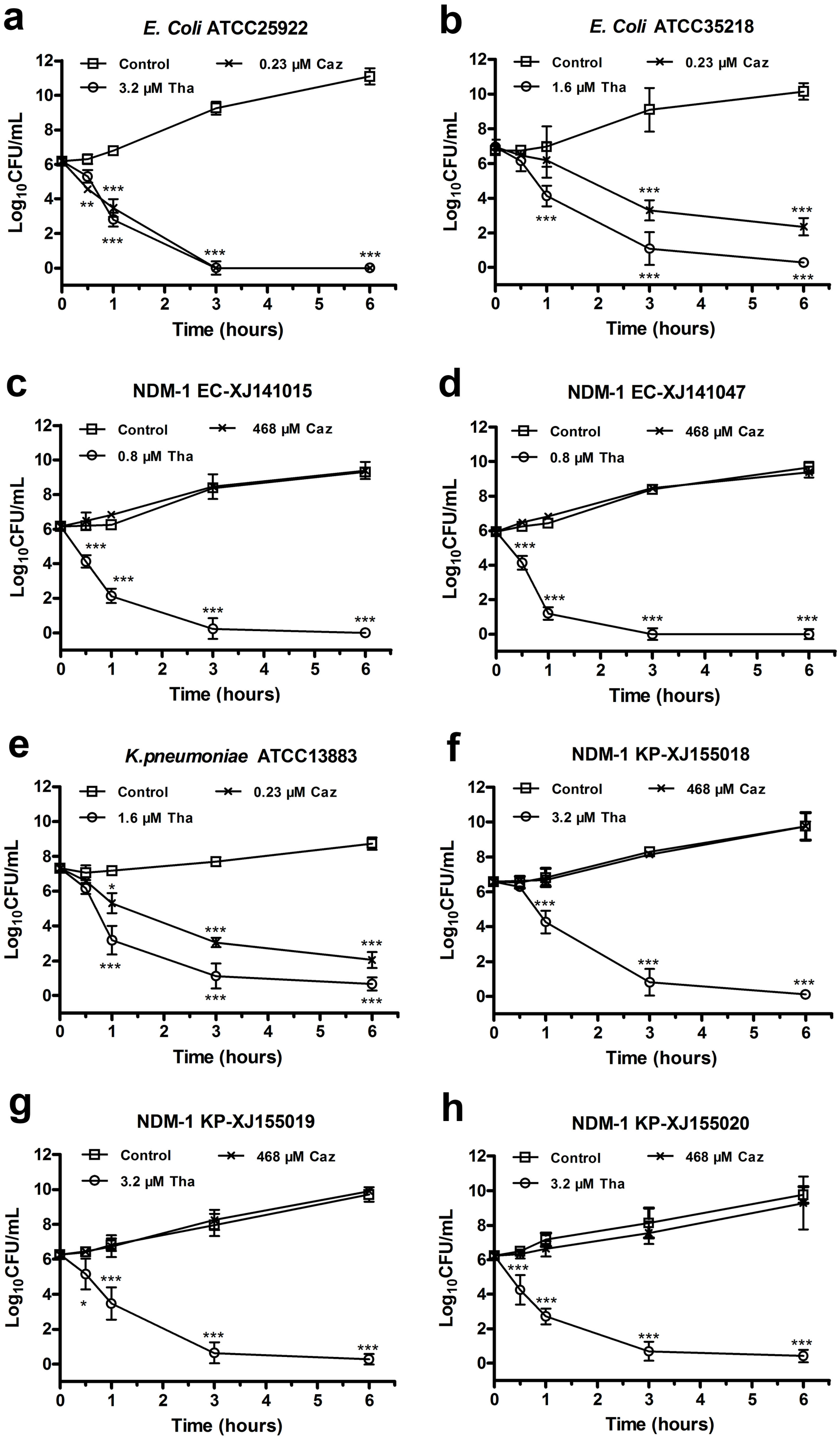

Supplementary Figure 2. Kill curves of thanatin and ceftazidime against clinical NDM-1 strains. NDM-1-producing *E. coli* (**a-d**) and *K. pneumoniae* (**e-h**) were treated with thanatin (Tha) and ceftazidime (Caz). Data are presented as the mean ± s.e.m. from three independent experiments. *$P < 0.05$, **$P < 0.01$, ***$P < 0.001$ versus control (determined by two-way ANOVA with Bonferroni's comparison). Source data are provided in Source Data file.

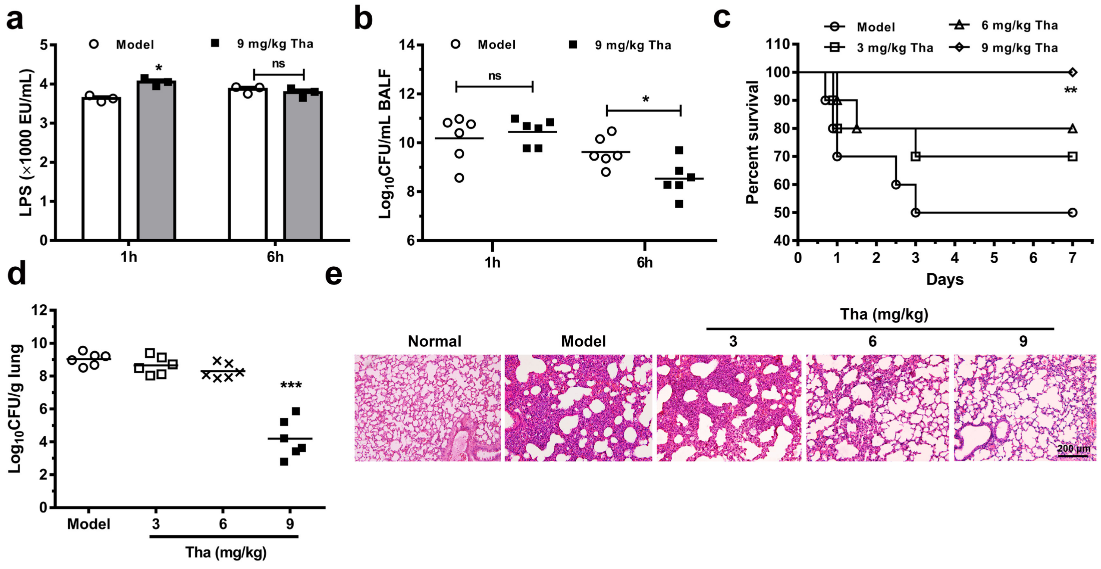

Supplementary Figure 3. Thanatin promotes the release of Ca^{2+} and LPS from the outer membrane of NDM-1-producing *K. pneumoniae* in vivo. a, b LPS levels (**a**) and bacterial loads (**b**) in the BALF of *K. pneumoniae* XJ155017-infected mice were analyzed at 1 and 6 h after 9 mg/kg thanatin treatment (n=6 per group). **c** Survival curves for the *K. pneumoniae* XJ155017 pneumonia model. BALB/c mice were intranasally administered with a sublethal dose of *K. pneumoniae* XJ155017 and treated with three doses of thanatin via intraperitoneal injection (n=10 per group). **d** Bacterial loads in the lungs of thanatin-treated *K. pneumoniae* XJ155017-infected mice were analyzed 24 h after infection (n=6 per group). **e** Lung morphology was examined with H&E staining in the pneumonia model after thanatin treatment. Scale bar = 200 μm. All data are shown as the mean ± s.e.m. *P* values were determined by two-way ANOVA (**a, b**), log-rank test (**c**), or one-way ANOVA with Bonferroni's comparison test (**d**). ns means not significant; $*P < 0.05$, $**P < 0.01$, $***P < 0.001$ versus model. Source data are provided in Source Data file.

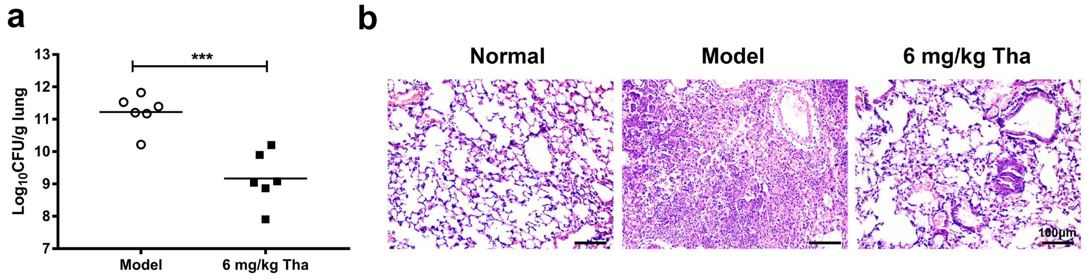

Supplementary Figure 4. Thanatin protects NDM-1-producing *E. coli*-infected pneumonia mice. a Bacterial loads in the lungs of thanatin-treated *E. coli* XJ141026-infected mice were analyzed 24 h after infection (n = 6 per group). Data are shown as the mean ± s.e.m. **b** Lung morphology was examined with H&E staining in the pneumonia model after thanatin treatment. Scale bar = 100 μm. *P* values were determined by two-tailed unpaired t test (**a**). ****P* < 0.001 versus model. Source data are provided in Source Data file.

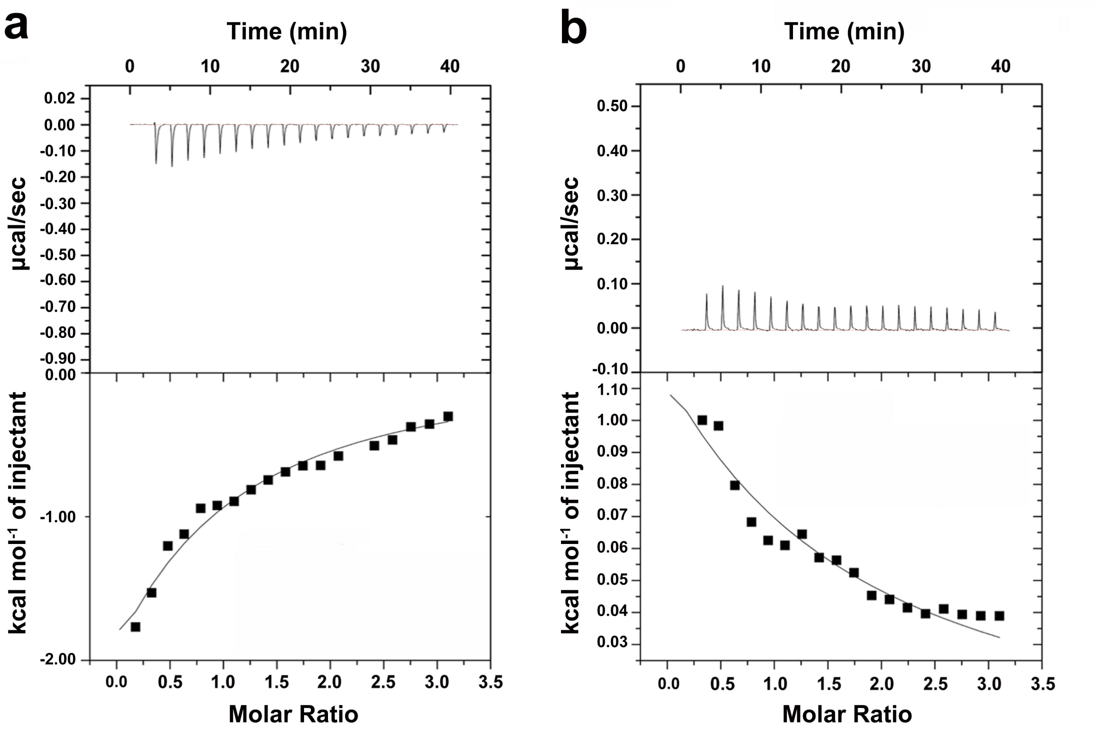

Supplementary Figure 5. ITC thermograms for the binding of LPS with Ca^{2+} (a), Mg^{2+} (b). The downward and upward peaks indicate the exothermic and endothermic process, respectively.

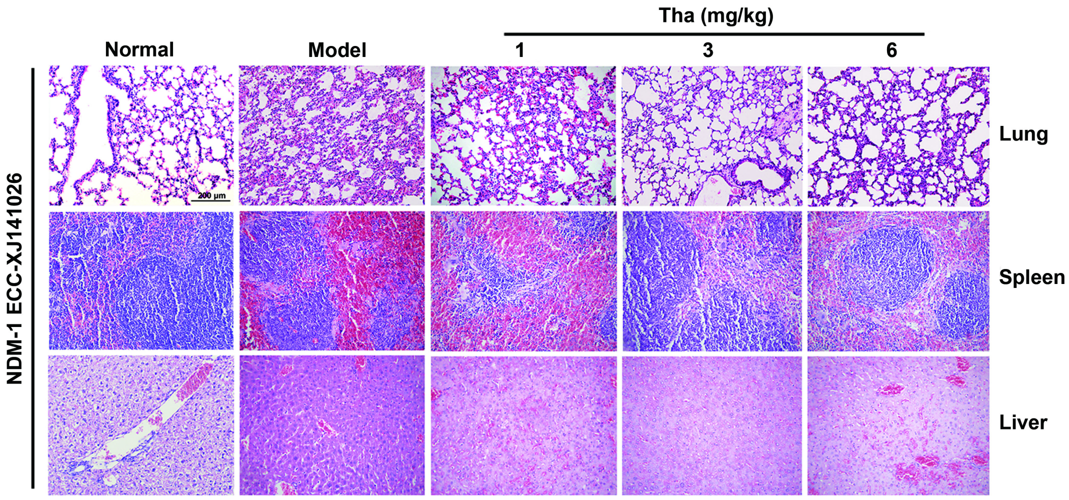

Supplementary Figure 6. H&E staining. Tissue morphology was examined with H&E staining in *E. coli* XJ141026 sepsis models after thanatin treatment. Scale bar = 200 μm.

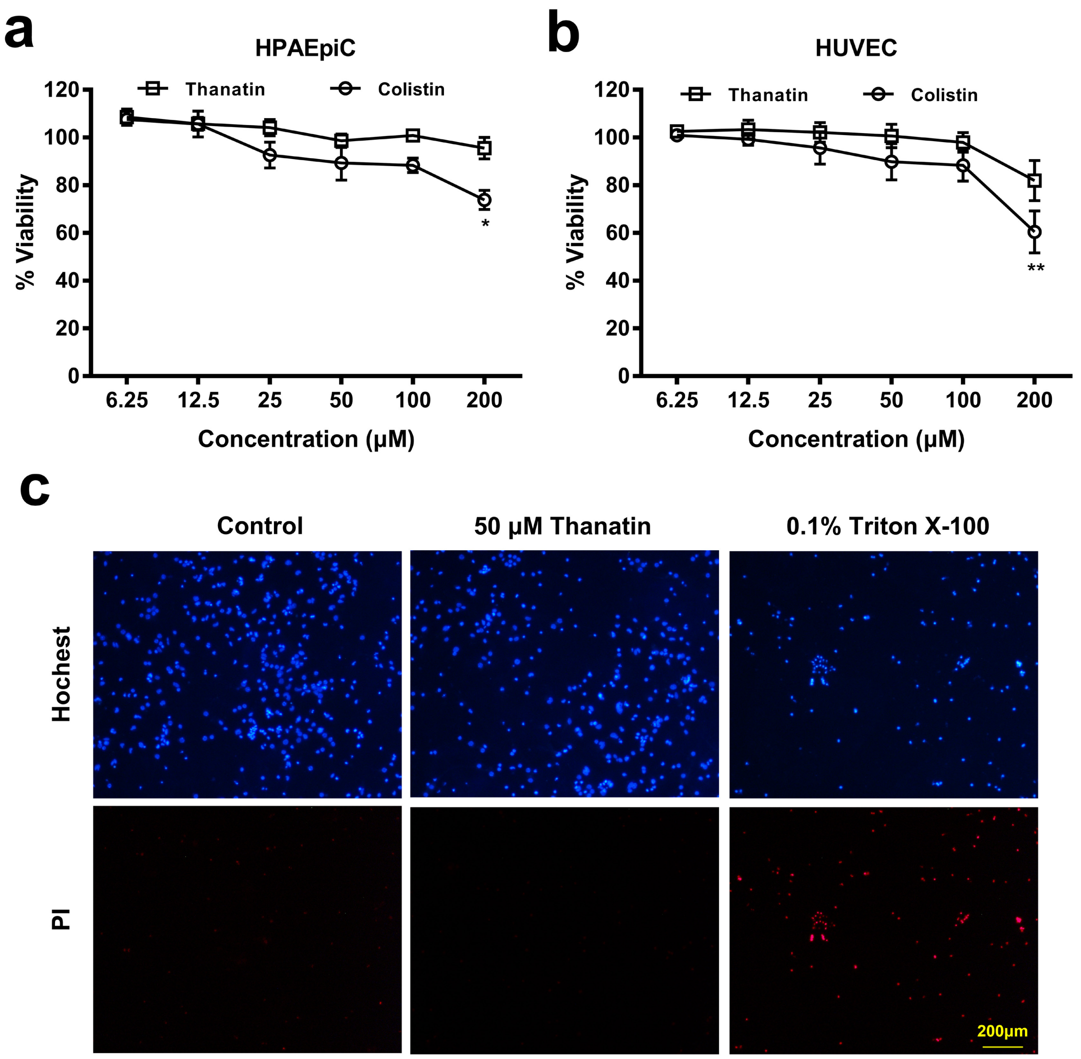

Supplementary Figure 7. Cytotoxicity of thanatin. a, b HPAEpiCs (**a**) and HUVECs (**b**) were treated with thanatin or colistin for 24 h, and cell viability was calculated on the basis of absorbance readings at 450 nm at 4 h after adding WST-8. **c** Representative photomicrographs of mouse neurons stained with Hoechst 33342 and PI fluorescent dye after exposure to 50 μM thanatin or 0.1% Triton X-100 for 24 h. Scale bar = 200 μm. Data are presented as the mean ± s.e.m. from at least three independent experiments. *$P < 0.05$, **$P < 0.01$ (determined by two-way ANOVA). Source data are provided in Source Data file.

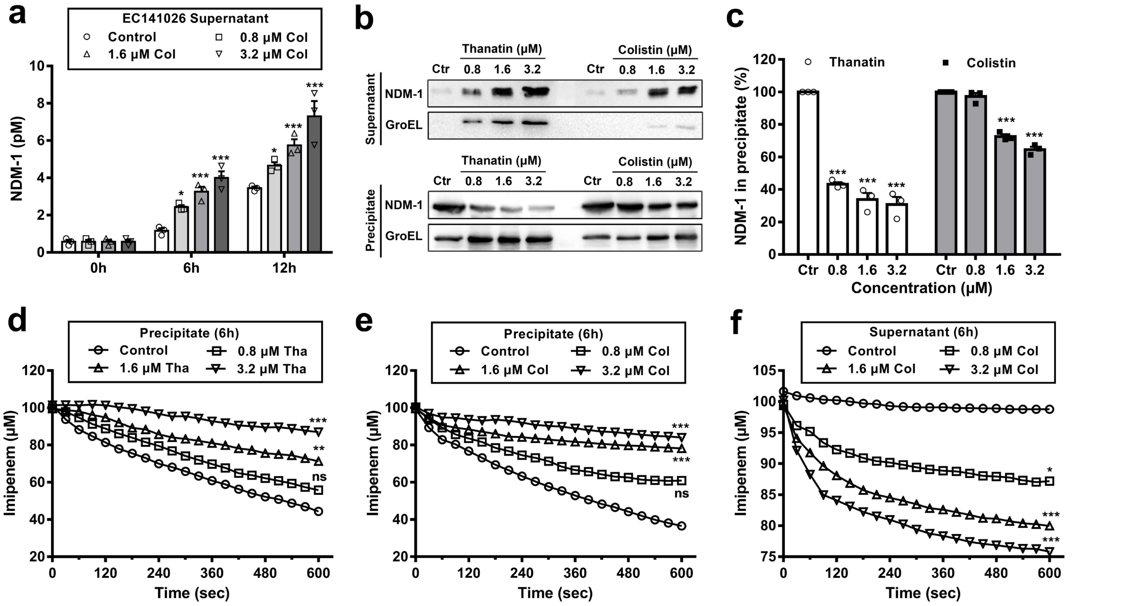

Supplementary Figure 8. NDM-1 protein levels and its hydrolytic activity to imipenem. **a** Increase in NDM-1 release to supernatants after treatment with colistin (Col). **b** Western blot analysis of NDM-1 in thanatin- and colistin-treated *E. coli* XJ141026 cell supernatants and precipitates for 6 h. GroEL is used as a loading control. **c** Relative expression of NDM-1 in thanatin- and colistin-treated *E. coli* XJ141026 cell precipitates for 6 h. **d** Hydrolytic effect of the cell precipitates obtained from thanatin-treated NDM-1 *E. coli* XJ141026 on imipenem. **e, f** Hydrolytic effect of the cell precipitates (**e**) and supernatants (**f**) obtained from colistin-treated NDM-1 *E. coli* XJ141026 on imipenem. The supernatants and cell precipitates were collected 6 h after incubation with thanatin or colistin. Data are presented as the mean ± s.e.m. from three independent experiments. *P* values were determined by two-way ANOVA (**a**, **d**–**f**), or by one-way ANOVA with Bonferroni's comparison test (**c**). ns means not significant; **P* < 0.05, ***P* < 0.01, ****P* < 0.001. Original western blot images for **b** are shown in **Supplementary Figure 13.** Source data are provided in Source Data file.

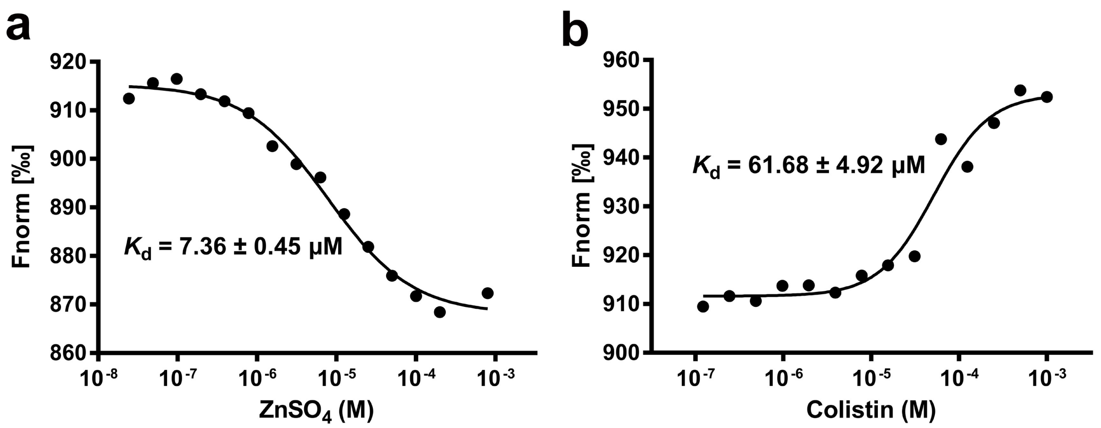

Supplementary Figure 9. MST assay. Interaction between RED-tris-NTA second-generation dye-labeled purified apo-NDM-1with $ZnSO_4$ (**a**) or colistin (**b**). Data are presented as the mean ± s.e.m. from three independent experiments. Source data are provided in Source Data file.

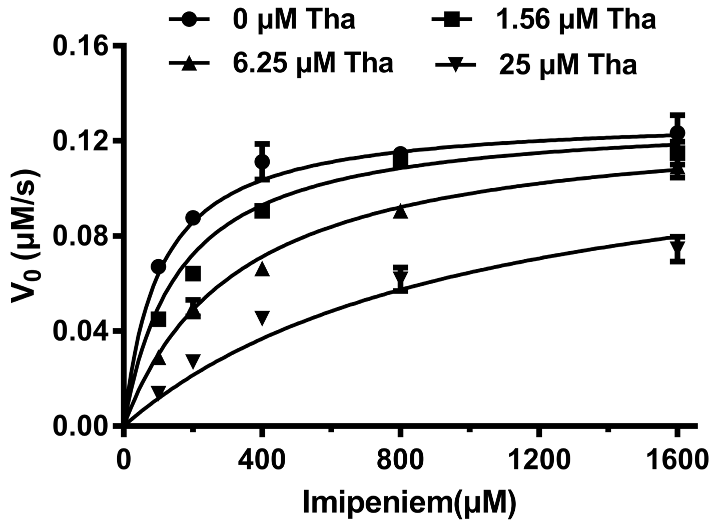

Supplementary Figure 10. In vitro inactivation of NDM-1 by thanatin. The initial rate of hydrolysis of imipenem (100, 200, 400, 800, and 1600 μM) by 1 nM NDM-1 was evaluated in the absence and presence of three concentrations of thanatin. The curves show the fits to the competitive inhibition model. Data are shown as the mean ± s.e.m. from three independent experiments. Source data are provided in Source Data file.

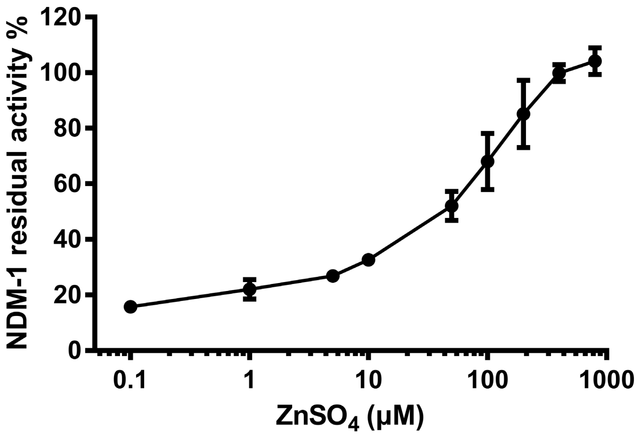

Supplementary Figure 11. Zn^{2+} restoration assays. NDM-1 activity inhibited by thanatin was gradually rescued with the increasing concentrations of Zn^{2+}. Data are shown as the mean ± s.e.m. from three independent experiments. Source data are provided in Source Data file.

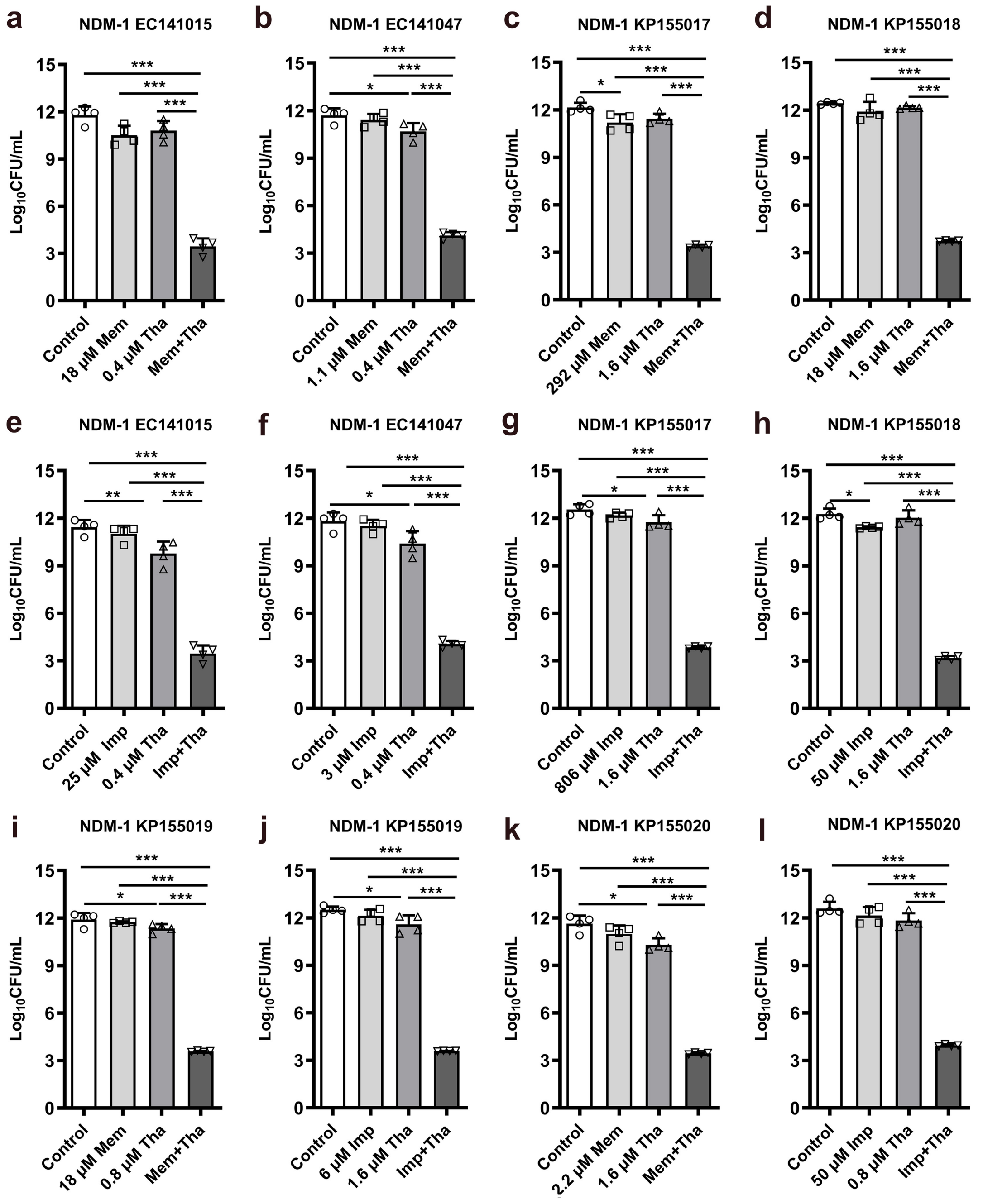

Supplementary Figure 12. Sub-MICs of thanatin reverse carbapenem resistance in vitro. Sub-MICs of thanatin restored the activity of meropenem (**a-d, i, k**) and imipenem (**e-h, j, l**) against NDM-1-producing *E. coli* and *K. pneumonia*. All data are shown as the mean ± s.e.m. from at least three independent experiments. **P* < 0.05, ***P* < 0.01, ****P* < 0.001 (determined by one-way ANOVA with Bonferroni's comparison). Source data are provided in Source Data file.

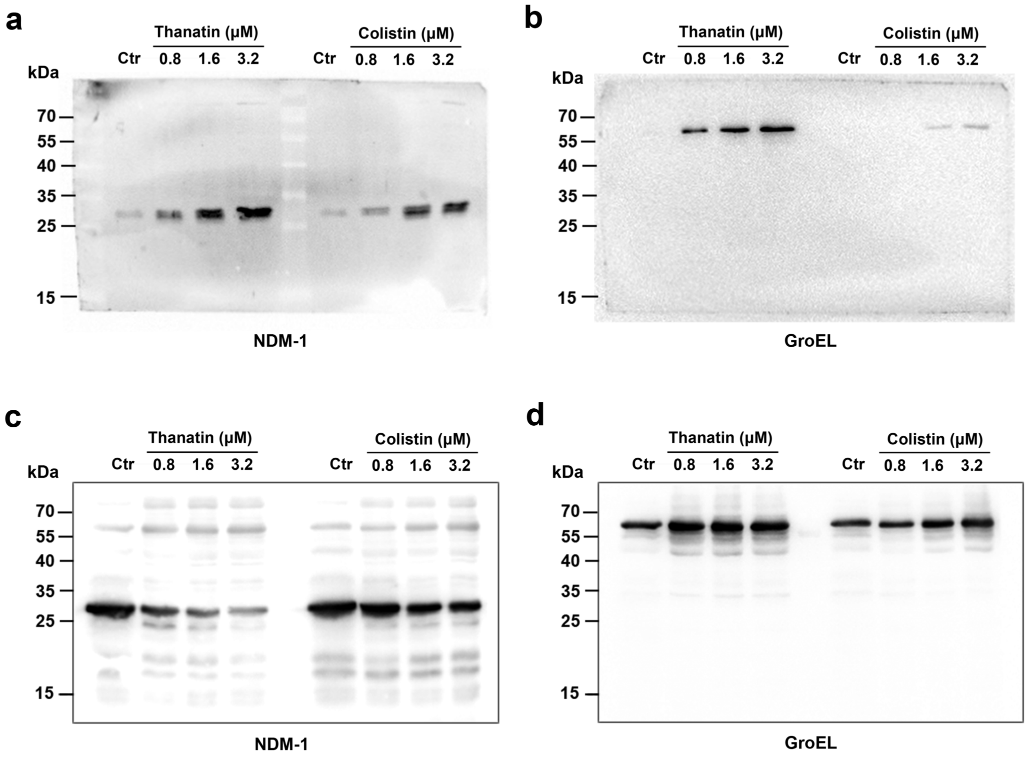

Supplementary Figure 13. Original western blot images for Supplementary Figure 8b. a, b Western blot analysis of NDM-1 (**a**) and GroEL (**b**) in cell supernatants of thanatin- and colistin-treated *E. coli* XJ141026. **c, d** Western blot analysis of NDM-1 (**c**) and GroEL (**d**) in cell precipitates of thanatin- and colistin-treated *E. coli* XJ141026.

Supplementary Table 1. Identification and antibiotic susceptibility of NDM-1-producing *E. coli* strains.

Antibiotics		Strains							
		E. coli ATCC25922		NDM-1 *E. coli* XJ141015		NDM-1 *E. coli* XJ141026		NDM-1 *E. coli* XJ141047	
**Imipenem**	**MIC**	<=1	（S）	>8	（R）	>8	（R）	>8	（R）
**Aztreonam**	**MIC**	<=2	（S）	>16	（R）	>16	（R）	>16	（R）
**Cefotaxime**	**MIC**	<=1	（S）	>32	（R）	>32	（R）	>32	（R）
**Ciprofloxacin**	**MIC**	<=0.5	（S）	>2	（R）	>2	（R）	<=0.5	（S）
**Ampicillin**	**MIC**	<=4	（S）	>16	（R）	>16	（R）	>16	（R）
**Meropenem**	**MIC**	<=1	（S）	>8	（R）	>8	（R）	>8	（R）
**Piperacillin**	**MIC**	<=4	（S）	>64	（R）	>64	（R）	>64	（R）
**Tetracycline**	**MIC**	<=2	（S）	<=2	（S）	>8	（R）	<=2	（S）
**Cefepime**	**MIC**	<=2	（S）	>16	（R）	>16	（R）	>16	（R）
**Moxifloxacin**	**MIC**	<=1	（S）	>4	（R）	>4	（R）	<=1	（S）
**Cotrimoxazole**	**MIC**	<=0.5/9.5	（S）	<=0.5/9.5	（S）	>2/38	（R）	<=0.5/9.5	（S）
**Aoxicillin/ clavulanic acid**	**MIC**	8/4	（S）	>16/8	（R）	>16/8	（R）	>16/8	（R）
**Chloramphenicol**	**MIC**	<=4	（S）	>16	（R）	<=4	（S）	<=4	（S）
**Gentamicin**	**MIC**	<=2	（S）	<=2	（S）	>8	（R）	>8	（R）
**Cefazolin**	**MIC**	<=4	（S）	>16	（R）	>16	（R）	>16	（R）
**Ampicillin/ sulbactam**	**MIC**	<=4/2	（S）	>16/8	（R）	>16/8	（R）	>16/8	（R）
**Piperacillin/ tazobactam**	**MIC**	<=4/2	（S）	>64/4	（R）	>64/4	（R）	>64/4	（R）
**Amikacin**	**MIC**	<=8	（S）	<=8	（S）	<=8	（S）	<=8	（S）
**Ceftazidime**	**MIC**	<=1	（S）	>16	（R）	>16	（R）	>16	（R）
**Levofloxacin**	**MIC**	<=1	（S）	>8	（R）	>8	（R）	<=1	（S）
**Cefoperazone/ sulbactam**	**KB**	—	—	7	（R）	7	（R）	7	（R）
**Nitrofurantoin**	**KB**	—	—	14	（R）	17	（R）	19	（S）
**Cefuroxime**	**KB**	—	—	7	（R）	7	（R）	7	（R）

R, resistant; S, sensitive; MIC, minimum inhibitory concentration; KB, Kirby Bauer disc diffusion method. The unit of MIC is “μg/mL”. The unit of KB is “mm”.

Supplementary Table 2. Identification and antibiotic susceptibility of NDM-1-producing *K. pneumonia* strains.

**Antibiotics**		**Strains**									
		K. pneumoniae ATCC13883		**NDM-1 *K. pneumoniae*** XJ155017		**NDM-1 *K. pneumoniae*** XJ155018		**NDM-1 *K. pneumoniae*** XJ155019		**NDM-1 *K. pneumoniae*** XJ155020	
**Imipenem**	**MIC**	<=1	(S)	>8	(R)	>8	(R)	>8	(R)	>8	(R)
**Aztreonam**	**MIC**	<=2	(S)	>16	(R)	>16	(R)	>16	(R)	>16	(R)
**Cefotaxime**	**MIC**	<=1	(S)	>32	(R)	>32	(R)	>32	(R)	>32	(R)
**Ciprofloxacin**	**MIC**	<=0.5	(S)	>2	(R)	>2	(R)	>2	(R)	>2	(R)
**Ampicillin**	**MIC**	16	(R)	>16	(R)	>16	(R)	>16	(R)	>16	(R)
**Meropenem**	**MIC**	<=1	(S)	>8	(R)	>8	(R)	>8	(R)	>8	(R)
**Piperacillin**	**MIC**	8	(S)	>64	(R)	>64	(R)	>64	(R)	>64	(R)
**Tetracycline**	**MIC**	<=2	(S)	4	(S)	>8	(R)	>8	(R)	>8	(R)
**Cefepime**	**MIC**	<=2	(S)	>16	(R)	>16	(R)	>16	(R)	>16	(R)
**Moxifloxacin**	**MIC**	<=1	(S)	>4	(R)	>4	(R)	>4	(R)	>4	(R)
**Cotrimoxazole**	**MIC**	=0.5/9.5	(S)	>1/19	(S)	>2/38	(R)	>2/38	(R)	>2/38	(R)
**Aoxicillin/ clavulanic acid**	**MIC**	<=4/2	(S)	>16/8	(R)	>16/8	(R)	>16/8	(R)	>16/8	(R)
**Chloramphenicol**	**MIC**	<=4	(S)	>16	(R)	>16	(R)	>16	(R)	>16	(R)
**Gentamicin**	**MIC**	<=2	(S)	<=2	(S)	<=2	(S)	<=2	(S)	<=2	(S)
**Cefazolin**	**MIC**	<=4	(S)	>16	(R)	>16	(R)	>16	(R)	>16	(R)
**Ampicillin/ sulbactam**	**MIC**	8/4	(S)	>16/8	(R)	>16/8	(R)	>16/8	(R)	>16/8	(R)
**Piperacillin/ tazobactam**	**MIC**	<=4/4	(S)	>64/4	(R)	>64/4	(R)	>64/4	(R)	>64/4	(R)
**Amikacin**	**MIC**	<=8	(S)	<=8	(S)	<=8	(S)	<=8	(S)	<=8	(S)
**Ceftazidime**	**MIC**	<=1	(S)	>16	(R)	>16	(R)	>16	(R)	>16	(R)
**Levofloxacin**	**MIC**	<=1	(S)	——	-	——	-	>8	(R)	>8	(R)
**Cefoperazone/ sulbactam**	**KB**	32	(S)	7	(R)	7	(R)	7	(R)	7	(R)
**Nitrofurantoin**	**KB**	21	(S)	7	(R)	9	(R)	10	(R)	9	(R)
**Cefuroxime**	**KB**	23	(S)	7	(R)	7	(R)	7	(R)	7	(R)

R, resistant; S, sensitive; MIC, minimum inhibitory concentration; KB, Kirby Bauer disc diffusion method. The unit of MIC is "µg/mL". The unit of KB is "mm".

Supplementary Table 3. Thermodynamic parameters for the binding of LPS with thanatin, Ca^{2+}, and Mg^{2+} were estimated by ITC.

	K_d (μM)	△H (Cal/mol)	△S (Cal/mol/deg)
**Thanatin + LPS**	1.09 ± 0.11	-6.46×10^3	5.62
**Ca^{2+} + LPS**	114.24 ± 26.05	-9.42×10^4	-2.98×10^2
**Mg^{2+} + LPS**	208.46 ± 52.28	2.83×10^6	9.50×10^3

Supplementary Table 4. Kinetic parameters for imipenem hydrolysis catalyzed by NDM-1.

**Kinetic parameters**	**Enzyme/substrate**
	**NDM-1/Imipenem**
K_m (μM)	103.80 ±10.34
V_{max} (μM·s^{-1})	0.13 ± 0.03
K_{cat} (s^{-1})	130.40 ± 2.67
