# Supplementary material for: The antimicrobial peptide thanatin disrupts the bacterial outer membrane and inactivates the NDM-1 metallo-β-lactamase
Source: Nat Commun. 2019 Aug 6;10:3517. doi: 10.1038/s41467-019-11503-3 (PMC6684654; doi:10.1038/s41467-019-11503-3)
Supplement: Supplementary file 3 — Reporting Summary [file 41467_2019_11503_MOESM3_ESM.pdf]

nature research

Corresponding author(s): Zheng Hou

Last updated by author(s): Jul 3, 2019

Reporting Summary

Nature Research wishes to improve the reproducibility of the work that we publish. This form provides structure for consistency and transparency in reporting. For further information on Nature Research policies, see Authors & Referees and the Editorial Policy Checklist.

Statistics

For all statistical analyses, confirm that the following items are present in the figure legend, table legend, main text, or Methods section.

n/a	Confirmed	
☐	☒	The exact sample size (*n*) for each experimental group/condition, given as a discrete number and unit of measurement
☐	☒	A statement on whether measurements were taken from distinct samples or whether the same sample was measured repeatedly
☐	☒	The statistical test(s) used AND whether they are one- or two-sided<br>*Only common tests should be described solely by name; describe more complex techniques in the Methods section.*
☐	☒	A description of all covariates tested
☐	☒	A description of any assumptions or corrections, such as tests of normality and adjustment for multiple comparisons
☐	☒	A full description of the statistical parameters including central tendency (e.g. means) or other basic estimates (e.g. regression coefficient) AND variation (e.g. standard deviation) or associated estimates of uncertainty (e.g. confidence intervals)
☐	☒	For null hypothesis testing, the test statistic (e.g. *F*, *t*, *r*) with confidence intervals, effect sizes, degrees of freedom and *P* value noted<br>*Give P values as exact values whenever suitable.*
☒	☐	For Bayesian analysis, information on the choice of priors and Markov chain Monte Carlo settings
☒	☐	For hierarchical and complex designs, identification of the appropriate level for tests and full reporting of outcomes
☐	☒	Estimates of effect sizes (e.g. Cohen's *d*, Pearson's *r*), indicating how they were calculated

Our web collection on statistics for biologists contains articles on many of the points above.

Software and code

Policy information about availability of computer code

Data collection	Syngistix, MO. Affinity Analysis, Gen5, Bioscreener, cellSens Entry, Tanon and FL solutions 2.0 softwares were used to collect data.
Data analysis	MO. Affinity Analysis and GraphPad Prism 5.0 was used to analyze data.

For manuscripts utilizing custom algorithms or software that are central to the research but not yet described in published literature, software must be made available to editors/reviewers. We strongly encourage code deposition in a community repository (e.g. GitHub). See the Nature Research guidelines for submitting code & software for further information.

Data

Policy information about availability of data

All manuscripts must include a data availability statement. This statement should provide the following information, where applicable:

- Accession codes, unique identifiers, or web links for publicly available datasets
- A list of figures that have associated raw data
- A description of any restrictions on data availability

The source data underlying Figs. 1a–d, 2, 3a–d, 4, 5, and 6b–f, and Supplementary Figs. 1, 2, 3a–d, 4a, 7a, b, 8a–f, 9, 10, 11, and 12 are provided in Source Data. The original data of the unprocessed blot and gel images are available in Source Data. The source data can also be found at https://doi.org/10.6084/m9.figshare.8481098.v1. All other data that support the findings of this study are available from the corresponding author upon reasonable request.

Field-specific reporting

Please select the one below that is the best fit for your research. If you are not sure, read the appropriate sections before making your selection.

☒ Life sciences ☐ Behavioural & social sciences ☐ Ecological, evolutionary & environmental sciences

For a reference copy of the document with all sections, see nature.com/documents/nr-reporting-summary-flat.pdf

Life sciences study design

All studies must disclose on these points even when the disclosure is negative.

Sample size	Sample sizes of at least three per group were chosen.
Data exclusions	There were no data exclusions.
Replication	All attempts at replication are successful.
Randomization	Mice were randomly assigned to experimental or control groups.
Blinding	Investigators were blinded to group allocation during data collection and analysis.

Reporting for specific materials, systems and methods

We require information from authors about some types of materials, experimental systems and methods used in many studies. Here, indicate whether each material, system or method listed is relevant to your study. If you are not sure if a list item applies to your research, read the appropriate section before selecting a response.

Materials & experimental systems

n/a	Involved in the study	
☐	☒	Antibodies
☐	☒	Eukaryotic cell lines
☒	☐	Palaeontology
☐	☒	Animals and other organisms
☒	☐	Human research participants
☒	☐	Clinical data

Methods

n/a	Involved in the study	
☒	☐	ChIP-seq
☒	☐	Flow cytometry
☒	☐	MRI-based neuroimaging

Antibodies

Antibodies used	Anti-NDM-1 antibodies were generated and validated as follows. New Zealand White rabbits were immunised with NDM-1. Once animal reaches the minimum titer of 1:50,000, collected serum and purified the antibody. Anti-GroEL antibody (ab82592) was purchased from Abcam.
Validation	Western and ELISA were used to validated the antibody, antibody purity were detected by SDS PAGE gels.

Eukaryotic cell lines

Policy information about cell lines

Cell line source(s)	HUVECs (ATCC-CRL1730); HPAEpiCs (ScienCell Research Lab., Catalog#3200, San Diego, CA)
Authentication	Both cell lines were identified by ATCC and ScienCell Research Lab when purchased. And we checked the cell lines by their morphological features.
Mycoplasma contamination	Both cell lines were tested to be mycoplasma-negative by the standard PCR method.
Commonly misidentified lines (See ICLAC register)	No commonly misidentified cell lines are used in this study.

Animals and other organisms

Policy information about studies involving animals; ARRIVE guidelines recommended for reporting animal research

Laboratory animals	Male BALB/c mice 8–10 weeks of age and weighing 18–22 g were obtained from the specific pathogen-free facility at the Fourth Military Medical University.
Wild animals	This study did not involve wild animals.
Field-collected samples	This study did not involve field-collected samples.
Ethics oversight	The experimental and animal care procedures were approved by the animal care and use committee of the Fourth Military Medical University.

Note that full information on the approval of the study protocol must also be provided in the manuscript.
